# Supplementary material for: Effective gene immunotherapy for melanoma utilizing an advanced in vivo electrotransfer system
Source: Mol Ther Oncol. 2025 Aug 14;33(3):201035. doi: 10.1016/j.omton.2025.201035 (PMC12419079; doi:10.1016/j.omton.2025.201035)
Supplement: Document S1. Figures S1–S4 [file mmc1.pdf]

OMTON, Volume *33*

Supplemental information

Effective gene immunotherapy for melanoma utilizing an advanced *in vivo* electrotransfer system

Loree C. Heller, Julie S. Singh, Jody C. Synowiec, Pavan Kumar Cherukuri, Nhat Phan, Guilan Shi, Mark J. Jaroszeski, Alex Otten, and Richard Heller

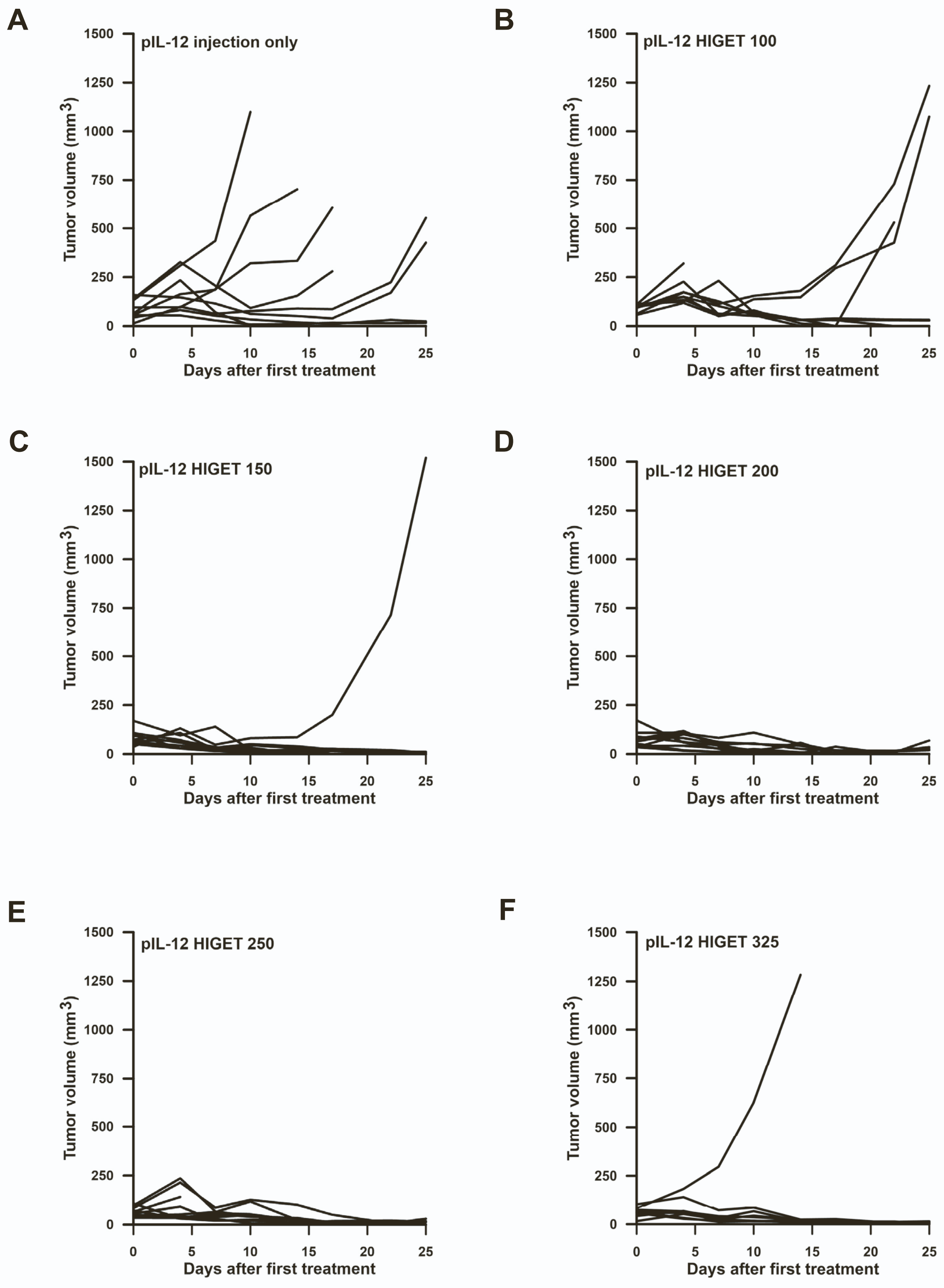

Figure S1. Individual tumor growth following **A,** pIL-12 injection only; **B,** pIL-12 injection plus HIGET 100 pulses; **C,** pIL-12 injection plus HIGET 150 pulses; **D,** pIL-12 injection plus HIGET 200 pulses; **E,** pIL-12 injection plus HIGET 250 pulses; **F** pIL-12 injection plus HIGET 325 pulses.

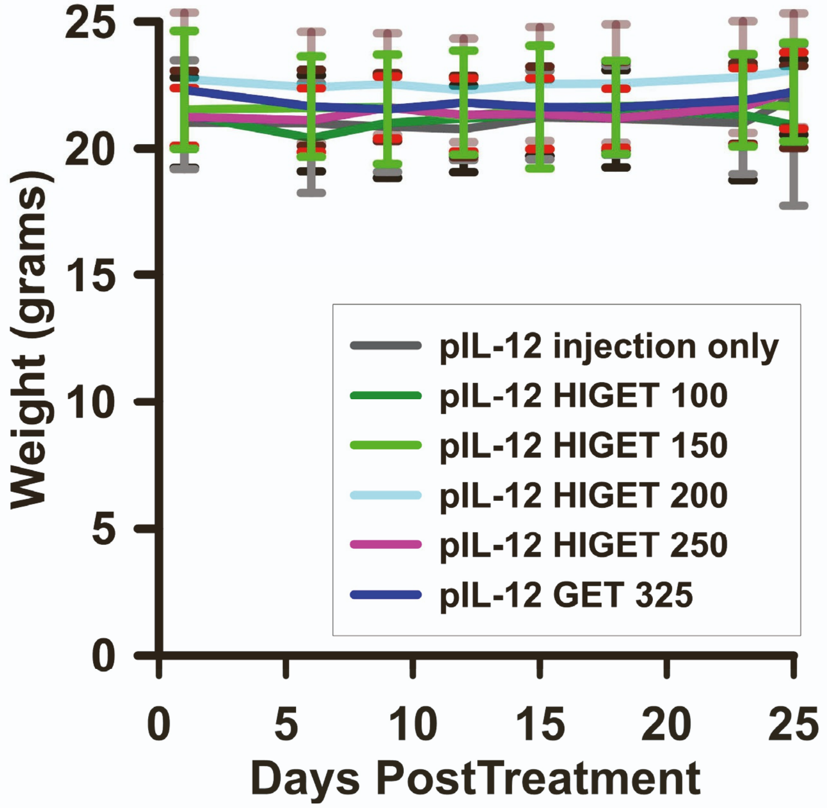

Figure S2. Body weight was not affected by HIGET delivery protocol. Numbers (100, 150, 200, 250, 325) indicate applied voltage (Table 1). Repeated measures ANOVA analysis indicated that weight did not significantly change across all dates and groups. pIL-12, pUMVC3-mIL-12; HIGET, heat and impedance gene electrotransfer.

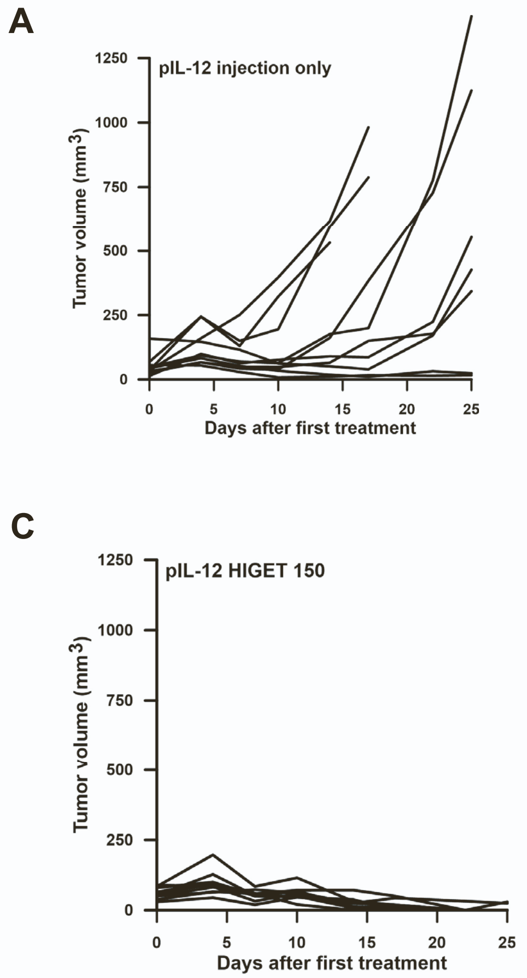

Figure S3. Individual tumor growth following **A,** pIL-12 injection only; **B,** pIL-12 injection plus GET 150 pulses; **C,** pIL-12 injection plus HIGET 150 pulses; **D,** pIL-12 injection plus GET 325 pulses.

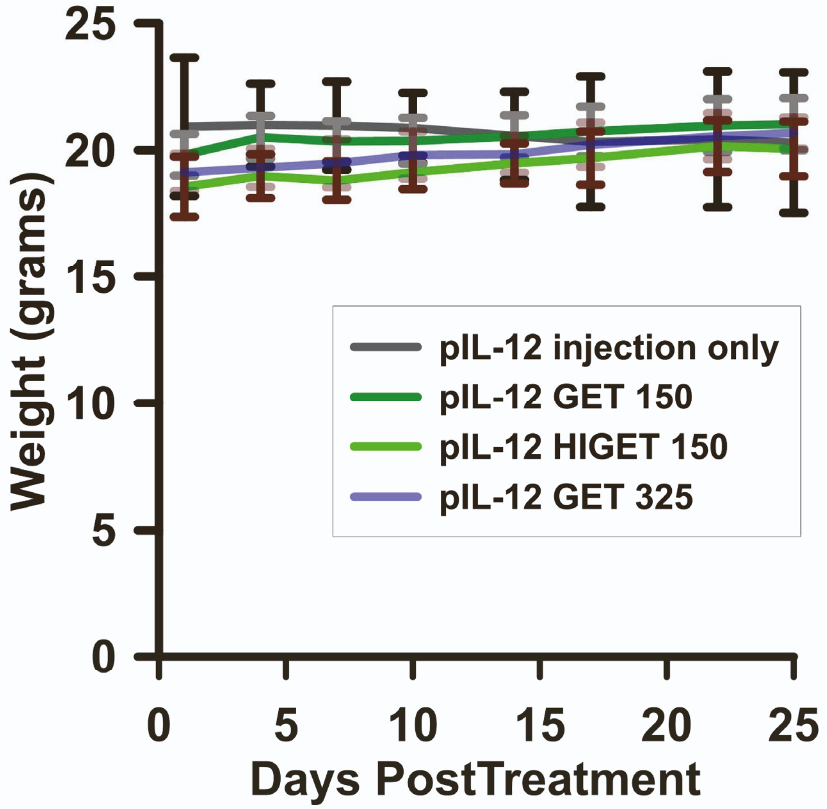

Figure S4. GET or HIGET plasmid delivery did not affect body weight. Numbers (150, 325) indicate applied voltage. Repeated measures ANOVA analysis indicated that weight did not significantly change across all dates and groups. pIL-12, pUMVC3-mIL12; GET, gene electrotransfer; HIGET, heat-and-impedance gene electrotransfer.
