# Supplementary material for: Effective gene immunotherapy for melanoma utilizing an advanced in vivo electrotransfer system
Source: Mol Ther Oncol. 2025 Aug 14;33(3):201035. doi: 10.1016/j.omton.2025.201035 (PMC12419079; doi:10.1016/j.omton.2025.201035)
Supplement: Document S2. Article plus supplemental information [file mmc2.pdf]

Molecular Therapy
Oncology
Original Article

Effective gene immunotherapy for melanoma utilizing an advanced *in vivo* electrotransfer system

Loree C. Heller,[1] Julie S. Singh,[1] Jody C. Synowiec,[1] Pavan Kumar Cherukuri,[1] Nhat Phan,[1] Guilan Shi,[1] Mark J. Jaroszeski,[1] Alex Otten,[1] and Richard Heller[1]

[1]Department of Medical Engineering, University of South Florida, Tampa, FL 33612, USA

Interleukin-12 (IL-12) is a potent immune stimulator that induces the proliferation and activation of natural killer (NK) and T cells and the secretion of interferon (IFN)-γ. While IL-12 is an effective anti-cancer therapy, high concentrations may produce unwanted adverse effects or result in immune suppression. We previously demonstrated that intratumor delivery of a plasmid encoding IL-12 with gene electrotransfer (GET) induced local and systemic tumor control with no detected adverse effects. However, the shortcomings of standard GET are that high voltage is required and the lack of a delivery completion signal. We recently developed an improved system that overcomes these issues by incorporating sophisticated electrodes, mild heat application, and real time tissue impedance monitoring. In this study, we validated this advanced electrotransfer system. We compared the therapeutic efficacy of intratumor IL-12 GET in B16-f10 mouse melanomas delivered by standard and advanced technologies. Delivery with both standard and advanced electrotransfer reduced the tumor specific growth rate and increased survival. Our results demonstrated that therapeutic *in vivo* gene delivery can be achieved with a less intense pulse regimen.

INTRODUCTION

Non-viral nucleic acid delivery methods comprise biological, chemical, and physical approaches.[1] While biological-based delivery has predominated, non-viral delivery has advanced substantially,[2] particularly with the success of nanoparticle vaccines.[3] Physical delivery modalities such as hydrodynamic delivery,[4] ultrasound,[5] and *in vivo* electroporation (EP) have also improved. EP is referred to as electrotransfer when used for molecular delivery.[6,7] Standard EP employs square wave pulses at predetermined field strengths, pulse lengths, and pulse numbers with limited electrode applicator designs. Despite these constraints, over 130 clinical trials have assessed EP for gene electrotransfer (GET), primarily for cancer therapy and viral vaccines.[8–11]

Interleukin-12 (IL-12) induces both innate and adaptive immunity, stimulating the proliferation and activation of T cells and natural killer (NK) cells and the secretion of interferon (IFN)-γ.[12,13] In both mouse studies and human clinical trials, IL-12 demonstrated significant anti-cancer effects.[14–16] When IL-12 was delivered as a recombinant protein, a 10% response rate was seen in melanoma patients. However, significant toxicity was noted in many of these studies and some trials were terminated ahead of schedule.[15,17] Modifying the therapy to a gene-based approach reduced adverse events while maintaining[18,19] or potentially exceeding[8] the antitumor activity of this cytokine.

When utilizing a gene-based immunotherapy approach, the level, location, and duration of transgene expression are critical variables. High expression may lead to over-stimulation of the immune system, leading to adverse events and/or immune suppression. In general, direct intratumor delivery resulting in localized expression can lead to reduced adverse events. Notably, localized IL-12 expression and delivery induces systemic tumor directed immune responses.[8,20–26]

The initial preclinical testing of intratumor delivery of a plasmid encoding the cytokine IL-12 effectively eradicated mouse melanomas and induced a vaccine effect, preventing formation of new tumors when mice were reinjected with B16-f10 melanoma cells.[27,28] A subsequent study demonstrated the significant reduction of lung metastases and the elimination of untreated tumors on the opposite flank.[20] Intracellular plasmid DNA activates DNA sensing, inducing an inflammatory response,[29] which potentiates IL-12 activity.[30] In clinical trials, IL-12 plasmid delivery to cutaneous or subcutaneous melanomas resulted in responses to both treated and untreated lesions with minimal to no adverse effects.[8] This therapeutic efficacy has been replicated in several clinical trials.[31,32]

Standard GET used for intratumor plasmid delivery includes the application of six 100 μs pulses at an applied voltage-to-distance ratio of 1,300 V/cm across a 9 mm electrode gap[8,27] and was administered in previous studies. In this study, we demonstrate effective

Received 15 April 2025; accepted 12 August 2025;
https://doi.org/10.1016/j.omton.2025.201035.

Correspondence: Richard Heller, Department of Medical Engineering, University of South Florida, Tampa, FL 33612, USA.
E-mail: rheller@usf.edu

Published by Elsevier Inc. on behalf of The American Society of Gene and Cell Therapy.
This is an open access article under the CC BY license (http://creativecommons.org/licenses/by/4.0/).

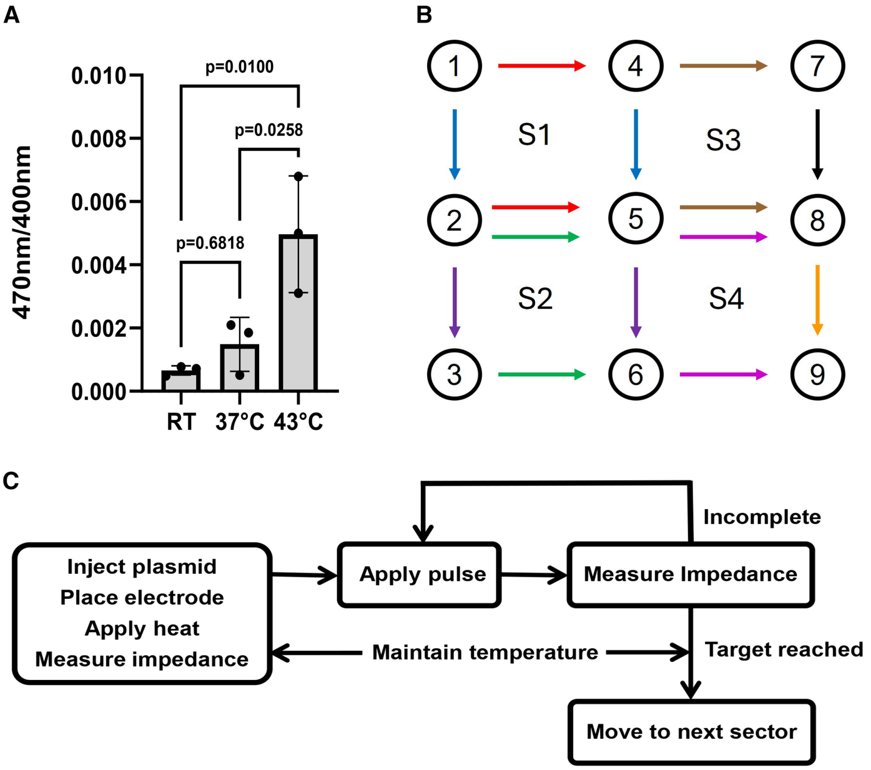

Figure 1. Intratumor HIGET protocol development

(A) B16-f10 melanoma cell membrane fluidity at room temperature, 37°C, and 43°C, $n = 3$ independent experiments. Data are presented as mean ± standard deviation analyzed by a one-way ANOVA followed by a Tukey-Kramer post-test with respect to room temperature control. (B) Electrode (1–9) and sector numbering (S1–S4). Pulses were applied in this order: sector 1, red then blue; sector 2, green then violet; sector 3, brown then black; sector 4, magenta then orange. (C) Functional schematic of a single electrode sector. RT, room temperature; S, electrode sector.

therapeutic *in vivo* plasmid delivery using an advanced EP technology, heat-and-impedance GET (HIGET), which addresses key limitations of standard GET technology for clinical use (submitted for publication; A.O., R.H., A. Hoff, T. Fawcett, and M.J.J.). Mild heat application during pulse application increases membrane fluidity (Figure 1A). We previously noted that increasing the temperature to 43°C augments electrotransfer delivery while higher temperatures (e.g. 45°C) increase the risk of tissue damage burning.[33] Consequently, the modified tissue electrical properties allow for a significant reduction in applied voltage while maintaining plasmid delivery and transgene expression.[33–35] To further enhance and control delivery, a sectored and independently addressable electrode configuration with a shortened electrode gap allows reduced pulse intensity (Figure 1B). Tissue impedance is monitored by averaging impedances in the frequency range of 1–3 kHz (hertz) in real time between each pulse to control the pulse number based on tissue- and individual- heterogeneity.[36,37] The procedure for a single electrode sector is outlined in Figure 1C. After plasmid injection, heat is applied. At this point, the initial impedance reading is measured. A pulse regimen is applied, and then impedance is remeasured. If an inadequate impedance decrease is detected, pulsing is repeated. When the target impedance is reached, pulsing moves on to a new electrode sector.

RESULTS

B16-f10 melanoma tumors were treated three times over one week (Figure 2A). Therapeutic efficacy was tested using a range of applied voltages for intratumor plasmid delivery. In these studies, no difference in tumor growth rate or survival were observed between intratumor pIL-12 injection alone or combined with HIGET pulses at a voltage to distance ratio of 100 V per 2.5 mm electrode gap (400 V/cm, HIGET 100, Table 1). However, delivery with HIGET applied voltages of 150, 200, 250, and 325 significantly reduced specific growth rate[38] (Figure 2B; Figure S1) and increased long-term survival to 80%–89% (Figure 2C) compared to simple intratumor pIL-12 injection. No markers of toxicity such as weight loss, change in coat appearance, general condition, or discomfort were observed. Body weight did not vary between groups (Figure S2).

We next quantified the tumor impedance changes for each pulse group. With a single exception, pulse application significantly reduced the tissue impedance in each electrode sector for each applied voltage (Figures 3A–3E). While the group receiving HIGET 100 pulse delivery saw no therapeutic benefit, tissue impedance significantly decreased to approximately 84% of the initial impedance. Delivery with HIGET 150 decreased tissue impedance to approximately 75%, HIGET 200 to 77%, HIGET 250 to 74%, and HIGET 325 to 71%. When compared to delivery with HIGET 100, the impedance significantly decreased in the remaining experimental groups in which a therapeutic benefit was observed (Table 2).

As previously observed,[20,39] pIL-12 delivery with standard GET 325 produced significant tumor growth delay and long-term complete regression in B16-f10 melanomas. A significant difference (p = 0.046) in survival was observed between groups receiving HIGET pulses of 100 and 150 applied volts (Figure 2C). We therefore compared the efficacy of pIL-12 delivery with HIGET 150, substandard GET 150 and standard GET 325 to pIL-12 injection only. Using the substandard GET 150 pulse regimen, neither the tumor-specific growth rate (Figure 4A; Figure S3) nor survival (Figure 4B) was significantly affected. However, pIL-12 delivery with standard GET 325 and HIGET 150 decreased the tumor growth rate (p <0.001) and increased survival (90%, p <0.001 and 100%, p <0.001,

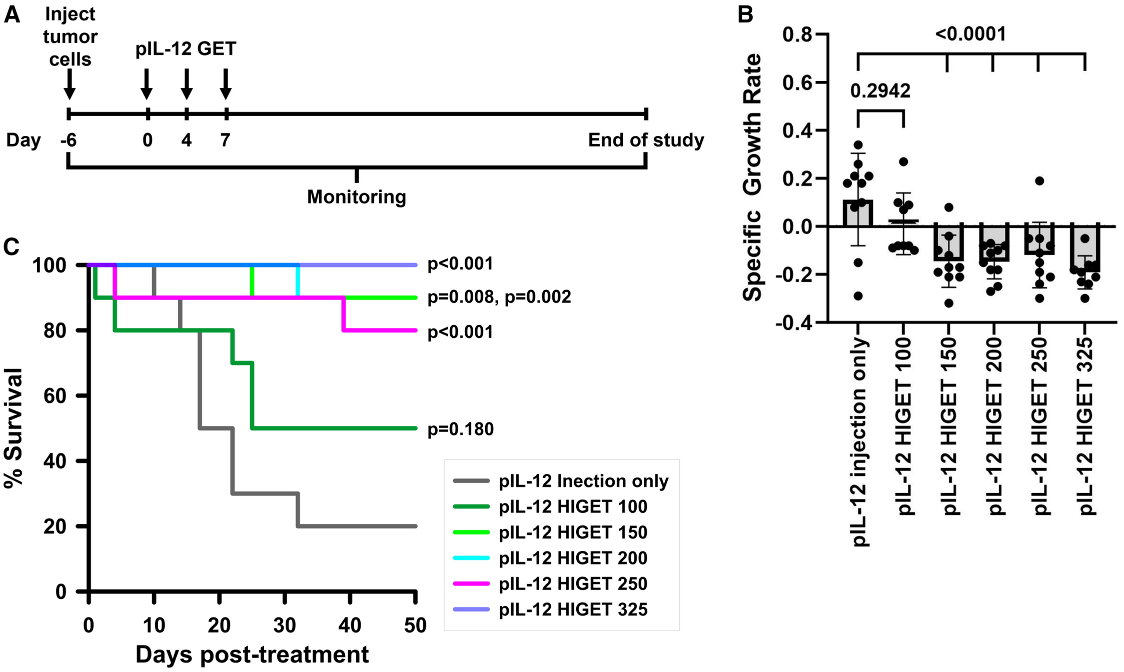

Figure 2. Intratumor HIGET protocol testing

(A) Timeline showing B16-f10 melanoma tumor generation, treatment, and monitoring. (B) Tumor growth rates are presented as mean ± standard deviation analyzed by a one-way ANOVA followed by a Tukey-Kramer post-test with respect to pIL-12 injection only control group. (C) Kaplan-Meier survival plot. Survival significance was determined by an overall Mantel-Cox log rank test followed by multiple comparisons using the Bonferroni method with respect to pIL-12 injection only control. Numbers (100, 150, 200, 250, and 325) indicate applied voltage (Table 1). pIL-12, pUMVC3-mIL-12; HIGET, heat and impedance gene electrotransfer.

respectively). Notably, no significant difference in survival (p = 0.3173) was observed between the standard GET 325 and the advanced HIGET 150 delivery groups. As with the previous experiment, no toxicity was observed. Once again, body weight did not vary between groups (Figure S4).

DISCUSSION

Relatively few treatment options for patients with advanced malignant melanoma are available.[40] Out of the more than 90,000 cases diagnosed each year, approximately 20% will have metastatic spread.[40,41] While multiple chemotherapy regimens have been tested for malignant melanoma, this approach does not increase overall survival.[42–44] Some success has been observed with immunotherapies.[45]

Recombinant IL-12 may be an effective melanoma therapy. However, serious adverse effects have been associated with this approach due to the multiple cycles of high concentration injections of recombinant IL-12 necessary to maintain therapeutic levels.[46] A gene-based approach can modulate the kinetics and potentially eliminate this toxicity. We previously demonstrated that intratumor GET effectively delivers plasmids encoding cytokines including IL-2,[47] granulocyte-macrophage colony-stimulating factor,[47] interferon-α,[48] IL-15,[49] and IL-12[20,27,28,39]. Each of these cytokines produced antitumor immunity and induced tumor regression, but IL-12 demonstrated robust local and systemic responses with no detectable toxicity.[50] This approach was translated to clinical trials, demonstrating a 14% complete response rate in both treated and untreated lesions in melanoma patients.[8,51] In these studies, the predominant adverse reaction was procedural pain (primarily grade 1) related to the application of the electric pulses.[8,50] In addition, minimal (5% of patients) systemic toxicities (grade 3 or higher) related to the therapy were observed.[8,50] IL-12 plasmid delivery with this advanced system replicates this lack of toxicity.

The current report investigated the efficacy of an advanced GET technology for intratumor IL-12 plasmid delivery for melanoma

Table 1. Pulse parameters

Group designation	Applied voltage (V)	Voltage/cm electrode distance (V/cm)
GET or HIGET 100	100	400
GET or HIGET 150	150	600
GET or HIGET 200	200	800
GET or HIGET 250	250	1,000
GET or HIGET 325	325	1,300

All pulses were 100 μs in length. The electrode gap was 0.25 cm. GET, standard gene electrotransfer with 6 pulses; HIGET, heat and impedance gene electrotransfer, pulse number varied based on the impedance values.

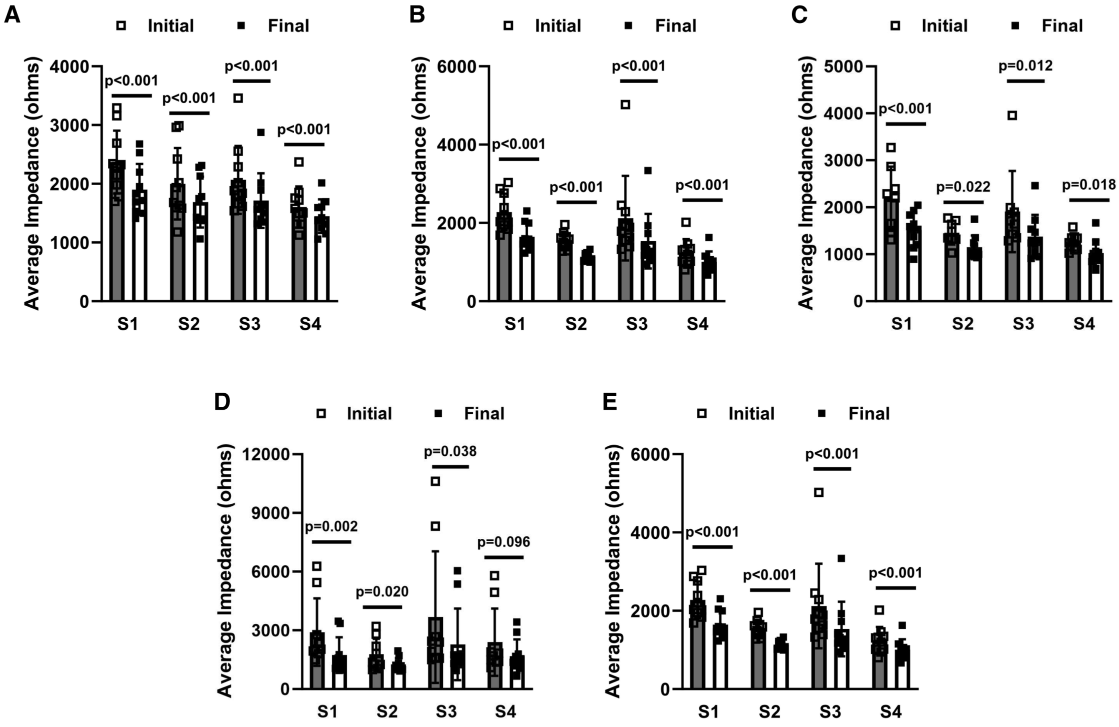

Figure 3. Initial and final tissue impedance changes in each electrode sector after pulsing

Initial and final comparisons are presented as mean ± standard deviation analyzed by paired Student's t test. (A) 100 applied volts. (B) 150 applied volts. (C) 200 applied volts. (D) 250 applied volts. (E) 325 applied volts. pIL-12, pUMVC3-mIL-12; HIGET, heat and impedance gene electrotransfer; S, electrode sector.

treatment. Overall, HIGET 100 pulse application significantly reduced tumor tissue impedance to approximately 85% of the initial impedance (Table 2) but offered no therapeutic benefit. No difference was detected between the remaining pulse groups (HIGET 150, 200, 250, and 325) in which a therapeutic benefit was observed, which reduced tumor tissue impedance to 71%–77% and varied significantly from HIGET 100. Clearly, a specific threshold in impedance decrease is needed to achieve a therapeutic benefit. It is possible that additional pulsing may further decrease the tissue impedance in this group, which may lead to an enhanced therapeutic outcome. It is also conceivable that HIGET 150 is the lowest voltage at this pulse width that can be applied to achieve this level of therapeutic outcome.

Real time tissue impedance monitoring compensated for tissue and individual electrical variations. Even in the highly homogenous mouse xenograft tumor model, individual variation was detected in each electrode sector. Although the differences in pulse number per electrode sector were not significant, this number varied from 3 to 6 to compensate for tissue heterogeneity. Pulse adjustment may be greater and more variable in spontaneous clinical tumors.

In this study, we validated an advanced *in vivo* electrotransfer system designed to reduce pulse intensity while maintaining therapeutic efficacy. Treatment related toxicity was not observed. Regardless of control or experimental group, tumor burden was the cause when animals succumbed. The incorporation of innovative electrodes, mild heat application and real time tissue impedance monitoring allowed a reduction of approximately 87% from 1,170 (applied voltage used in previous preclinical and clinical trials) to 150 applied volts. We were able to replicate the efficacy of a tumor-directed gene therapy using a milder and directed pulse regimen. Previous work[27,39] with standard GET technology demonstrated that pulses alone do not affect tumor growth, while empty vector electrotransfer is known to have antitumor effects.[29,52–55] Future studies will document the contributions of these individual therapeutic components. Since this initial study demonstrates effectiveness in a melanoma model, we anticipate eventually initiating clinical trials in stage 3 melanoma patients. Ultimately, most accessible solid tumors should benefit from this approach.

MATERIALS AND METHODS

Plasmid

Plasmid pUMVC3-mIL12 (pIL-12, University of Michigan Biomedical Research Core Facilities, Ann Arbor, MI, USA) was commercially prepared (Aldevron LLC, Fargo, ND, USA) and suspended in sterile saline. Endotoxin levels were confirmed to be <0.1 EU/μg.

Table 2. Post-pulse impedance levels within each electrode sector

	Mean (ohms)	Median (ohms)	Range (ohms)	Final % change	p
HIGET 100 (n = 40)					
Initial	2,010	1,838	1,126–3,458	–	–
Final	1,688	1,594	1,533–2,875	84.9 ± 0.52	–
HIGET 150 (n = 40)					
Initial	1,802	1,636	803–5,024	–	–
Final	1,332	1,208	662–1,332	75.0 ± 0.05	$p = 0.002$
HIGET 200 (n = 32)					
Initial	1,702	1,469	1,022–3,958	–	–
Final	1,262	1,177	842–2,462	76.6 ± 0.14	$p = 0.025$
HIGET 250 (n = 36)					
Initial	2,686	1,898	966–10,619	–	–
Final	1,819	1,322	923–6,045	74.3 ± 0.16	$p < 0.001$
HIGET 325 (n = 36)					
Initial	2,043	1,893	896–3,684	–	–
Final	1,387	1,300	815–2,266	71.2 ± 0.14	$p < 0.001$

Final percentage impedance based on paired values. Percentage data are presented as mean ± standard deviation analyzed by a one-way ANOVA followed by a Tukey-Kramer post-test with respect to HIGET 100. HIGET, heat and impedance gene electrotransfer.

Cells

B16-f10 mouse melanoma cells (CRL-6475, American Type Culture Collection, Manassas, VA, USA) were cultured in McCoy's medium (Corning, Thermo Fisher Scientific, USA) supplemented with 10% fetal bovine serum (Gibco, Waltham, MA, USA) and 1% penicillin-streptomycin in a 5% CO_2 humidified incubator at 37°C. Mouse short tandem repeat profile and interspecies contamination tests were performed by IDEXX BioAnalytics (Columbia, MO, USA. The cells were negative for mycoplasma infection using the Myco-Sniff PCR Detection Kit (MP Biochemicals, Irvine, CA, USA).

Membrane fluidity

Cells were labeled with a membrane fluidity kit (Abcam, Cambridge, UK) per manufacturer's instructions. Room temperature (RT) cells were heated to 37°C or 43°C for one minute as determined by a thermocouple meter (HH801A, Omega Engineering, Stamford, CT, USA). The ratio between homodimer (470 nm) and monomer (405 nm) fluorescence emissions was determined (CLARIOStar, BMG Labtech, Cary, NC, USA) after subtraction of the background of unlabeled cells.

Tumor induction

All animal procedures were approved by the University of South Florida Institutional Animal Care and Use Committee. Briefly, 10^6 B16-f10 melanoma cells in 50 μL phosphate buffered saline were injected subcutaneously in the left flank of female C57Bl/6 mice (7–8 weeks old) (Jackson Laboratories, Bar Harbor, ME) and allowed to grow for six to eight days to a diameter of 4–6 mm (day 0).

Tumor treatment

Animals with palpable tumors were randomized into groups using Studylog Animal Study Workflow Software (South San Francisco, CA, USA). Tumors were treated on days 0, 4, and 7 (Figure 2A). Mice were anesthetized by inhalation of 3% isoflurane in oxygen and tumors were injected with 50 μg pUMVC3-mIL-12 in 25 μL saline. Pulses were applied as described (Table 1) using standard GET or advanced HIGET technology.

Impedance measurements

Impedance measurements were performed using sinusoids of discrete frequency applied one after another to the tissue through the custom electrode. A total of 11 frequencies were measured between 1 and 3 kHz in steps of 200 Hz, with the total measurement taking approximately 200 ms between EP pulses. The sinusoids were applied by, and the resulting currents measured by, an AD5933 network analyzer chip (Analog Devices, Wilmington, MA, USA) and converted to complex impedances at each of the measured frequencies. The norm of each impedance was taken as the magnitude of the complex impedance, $Z = \sqrt{R^2+I^2}$, where R is the real part and I is the imaginary part of the impedance. The mean of the norms of all frequencies was calculated for each measurement. Prior to gene delivery, an impedance measurement was taken in this way for all sectors of the electrode. These means were stored as a starting impedance for each respective sector. Following each pulse in sector one, the impedance was again collected as previously described and compared to the respective starting impedance. If the new post-pulse impedance had not adequately changed, the sector was again pulsed and measured for changes in impedance. If at any point the impedance changed by a threshold amount, pulsing was stopped in that sector and the sequence moved to treatment of the next sector. Each sector was measured and treated in this way until all four sectors were treated.

Tumor growth and mouse survival

Tumor growth or regression was measured twice weekly using a digital caliper. Tumor volume was calculated by the standard formula $v = ab^2 \times 0.5236$, where a is the longest diameter, and b is the next longest diameter perpendicular to a. Specific tumor growth rates between control and experimental groups were compared as described.[38] In cases where regression was observed, the values were calculated using the volumes and times to half the tumor size (negative tumor growth).

In the case of continued tumor growth or tumor recurrence, the animal was considered incurable and humanely euthanized when the tumor volume reached 1,000 mm.[3]

Statistical analysis

Statistical analyses were carried out using Graphpad Prism 10 or Graphpad InStat software (GraphPad Software, Boston, MA,

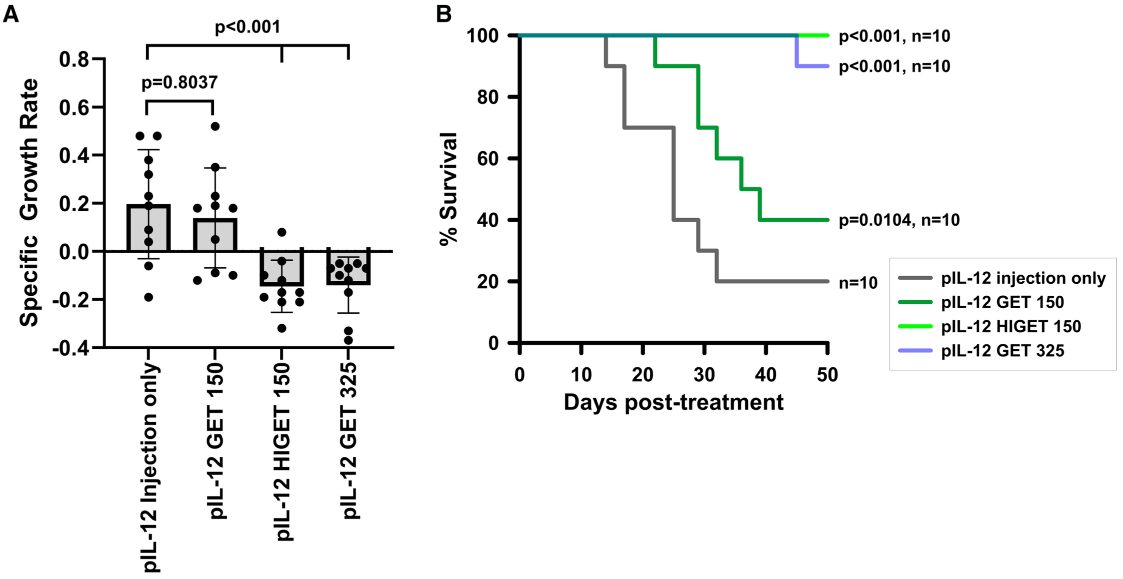

Figure 4. Therapeutic testing of intratumor HIGET delivery in B16-f10 melanoma mouse model

(A) Tumor growth rates are presented as mean ± standard deviation analyzed by a one-way ANOVA followed by a Tukey-Kramer post-test with respect to pIL-12 injection only control group. (B) Kaplan Meier survival plot. Survival significance was determined by an overall Mantel-Cox log rank test followed by multiple comparisons using the Bonferroni method with respect to pIL-12 injection only control. Numbers (150 and 325) indicate applied voltage (Table 1). pIL-12, pUMVC3-mIL12; GET, gene electrotransfer; HIGET, heat-and-impedance gene electrotransfer.

USA). Values are presented as the mean ± SD. Differences between two groups were determined by Student's t test. For more than two groups, one-way ANOVA was followed by a Tukey-Kramer post-test. Survival significance was determined by an overall Mantel-Cox log rank test followed by multiple comparisons using the Bonferroni method. In each analysis, a p value of less than 0.05 was considered statistically significant.

DATA AND CODE AVAILABILITY

Data were generated by the authors and available on request from the corresponding author.

ACKNOWLEDGMENTS

This research was supported, in part, by the Extramural Research Program of the NIH/NCI grant R01 CA186730 and by the Department of Medical Engineering. The funders had no role in study design, collection of data, decision to publish, or in preparation of this manuscript.

AUTHOR CONTRIBUTIONS

L.C.H., formal analysis, methodology, resources, validation, visualization, writing – original draft, and writing – review & editing. J.S.S., investigation, validation, and writing – review & editing. J.C.S., investigation, validation, and writing – review & editing. P.K.C., investigation and writing – review & editing. N.P., investigation and writing – review & editing. G.S., investigation, validation, and writing – review & editing. M.J.J., conceptualization, funding acquisition, methodology, project administration, resources, supervision, and writing – review & editing. A.O., investigation and writing – review & editing. R.H., conceptualization, funding acquisition, methodology, project administration, resources, supervision, and writing – review & editing. All authors read and approved the manuscript.

DECLARATION OF INTERESTS

R.H. and M.J.J. are inventors on patents that cover some of the work reported in this article. R.H. and M.J.J. have an ownership interest in MMD Technologies Corp., which has licensed those patents from the University of South Florida and Old Dominion University.

SUPPLEMENTAL INFORMATION

Supplemental information can be found online at https://doi.org/10.1016/j.omton.2025.201035.

REFERENCES

1. Sayed, N., Allawadhi, P., Khurana, A., Singh, V., Navik, U., Pasumarthi, S.K., Khurana, I., Banothu, A.K., Weiskirchen, R., and Bharani, K.K. (2022). Gene therapy: Comprehensive overview and therapeutic applications. Life Sci. *294*, 120375. https://doi.org/10.1016/j.lfs.2022.120375.
2. Sheridan, C. (2023). Why gene therapies must go virus-free. Nat. Biotechnol. *41*, 737–739. https://doi.org/10.1038/s41587-023-01824-6.
3. Zhang, G., Tang, T., Chen, Y., Huang, X., and Liang, T. (2023). mRNA vaccines in disease prevention and treatment. Signal Transduct. Target. Ther. *8*, 365. https://doi.org/10.1038/s41392-023-01579-1.
4. Suda, T., and Liu, D. (2007). Hydrodynamic gene delivery: its principles and applications. Mol. Ther. *15*, 2063–2069. https://doi.org/10.1038/sj.mt.6300314.
5. Song, S., Shen, Z., Chen, L., Brayman, A.A., and Miao, C.H. (2011). Explorations of high-intensity therapeutic ultrasound and microbubble-mediated gene delivery in mouse liver. Gene Ther. *18*, 1006–1014. https://doi.org/10.1038/gt.2011.34.
6. Heller, R., Jaroszeski, M., Atkin, A., Moradpour, D., Gilbert, R., Wands, J., and Nicolau, C. (1996). In vivo gene electroinjection and expression in rat liver. FEBS Lett. *389*, 225–228. https://doi.org/10.1016/0014-5793(96)00590-x.
7. Young, J.L., and Dean, D.A. (2015). Electroporation-mediated gene delivery. Adv. Genet. *89*, 49–88. https://doi.org/10.1016/bs.adgen.2014.10.003.
8. Daud, A.I., DeConti, R.C., Andrews, S., Urbas, P., Riker, A.I., Sondak, V.K., Munster, P.N., Sullivan, D.M., Ugen, K.E., Messina, J.L., and Heller, R. (2008). Phase I Trial of Interleukin-12 Plasmid Electroporation in Patients With Metastatic Melanoma. J. Clin. Oncol. *26*, 5896–5903. https://doi.org/10.1200/JCO.2007.15.6794.

9. Low, L., Mander, A., McCann, K., Dearnaley, D., Tjelle, T., Mathiesen, I., Stevenson, F., and Ottensmeier, C.H. (2009). DNA vaccination with electroporation induces increased antibody responses in patients with prostate cancer. Hum. Gene Ther. *20*, 1269–1278. https://doi.org/10.1089/hum.2009.067.

10. Vasan, S., Hurley, A., Schlesinger, S.J., Hannaman, D., Gardiner, D.F., Dugin, D.P., Boente-Carrera, M., Vittorino, R., Caskey, M., Andersen, J., et al. (2011). In vivo electroporation enhances the immunogenicity of an HIV-1 DNA vaccine candidate in healthy volunteers. PLoS One *6*, e19252. https://doi.org/10.1371/journal.pone.0019252.

11. Kalams, S.A., Parker, S.D., Elizaga, M., Metch, B., Edupuganti, S., Hural, J., De Rosa, S., Carter, D.K., Rybczyk, K., Frank, I., et al. (2013). Safety and Comparative Immunogenicity of an HIV-1 DNA Vaccine in Combination with Plasmid Interleukin 12 and Impact of Intramuscular Electroporation for Delivery. J. Infect. Dis. *208*, 818–829. https://doi.org/10.1093/infdis/jit236.

12. Trinchieri, G. (2003). Interleukin-12 and the regulation of innate resistance and adaptive immunity. Nat. Rev. Immunol. *3*, 133–146. https://doi.org/10.1038/nri1001.

13. Del Vecchio, M., Bajetta, E., Canova, S., Lotze, M.T., Wesa, A., Parmiani, G., and Anichini, A. (2007). Interleukin-12: biological properties and clinical application. Clin. Cancer Res. *13*, 4677–4685. https://doi.org/10.1158/1078-0432.CCR-07-0776.

14. Brunda, M.J., Luistro, L., Rumennik, L., Wright, R.B., Wigginton, J.M., Wiltrout, R.H., Hendrzak, J.A., and Palleroni, A.V. (1996). Interleukin-12: murine models of a potent antitumor agent. Ann. N. Y. Acad. Sci. *795*, 266–274. https://doi.org/10.1111/j.1749-6632.1996.tb52676.x.

15. Atkins, M.B., Robertson, M.J., Gordon, M., Lotze, M.T., DeCoste, M., DuBois, J.S., Ritz, J., Sandler, A.B., Edington, H.D., Garzone, P.D., et al. (1997). Phase I evaluation of intravenous recombinant human interleukin 12 in patients with advanced malignancies. Clin. Cancer Res. *3*, 409–417.

16. Nagai, H., Oniki, S., Fujiwara, S., Yoshimoto, T., and Nishigori, C. (2010). Antimelanoma immunotherapy: clinical and preclinical applications of IL-12 family members. Immunotherapy *2*, 697–709. https://doi.org/10.2217/imt.10.46.

17. Cavallo, F., Di Carlo, E., Butera, M., Verrua, R., Colombo, M.P., Musiani, P., and Forni, G. (1999). Immune events associated with the cure of established tumors and spontaneous metastases by local and systemic interleukin 12. Cancer Res. *59*, 414–421.

18. Heinzerling, L., Burg, G., Dummer, R., Maier, T., Oberholzer, P.A., Schultz, J., Elzaouk, L., Pavlovic, J., and Moelling, K. (2005). Intratumoral injection of DNA encoding human interleukin 12 into patients with metastatic melanoma: clinical efficacy. Hum. Gene Ther. *16*, 35–48.

19. Mahvi, D.M., Henry, M.B., Albertini, M.R., Weber, S., Meredith, K., Schalch, H., Rakhmilevich, A., Hank, J., and Sondel, P. (2007). Intratumoral injection of IL-12 plasmid DNA–results of a phase I/IB clinical trial. Cancer Gene Ther. *14*, 717–723. https://doi.org/10.1038/sj.cgt.7701064.

20. Lucas, M.L., and Heller, R. (2003). IL-12 gene therapy using an electrically mediated nonviral approach reduces metastatic growth of melanoma. DNA Cell Biol. *22*, 755–763. https://doi.org/10.1089/104454903322624966.

21. Ellmark, P., Mangsbo, S.M., Furebring, C., Norlén, P., and Tötterman, T.H. (2017). Tumor-directed immunotherapy can generate tumor-specific T cell responses through localized co-stimulation. Cancer Immunol. Immunother. *66*, 1–7. https://doi.org/10.1007/s00262-016-1909-3.

22. Aznar, M.A., Tinari, N., Rullán, A.J., Sánchez-Paulete, A.R., Rodriguez-Ruiz, M.E., and Melero, I. (2017). Intratumoral Delivery of Immunotherapy-Act Locally, Think Globally. J. Immunol. *198*, 31–39. https://doi.org/10.4049/jimmunol.1601145.

23. Nguyen, K.G., Vrabel, M.R., Mantooth, S.M., Hopkins, J.J., Wagner, E.S., Gabaldon, T.A., and Zaharoff, D.A. (2020). Localized Interleukin-12 for Cancer Immunotherapy. Front. Immunol. *11*, 575597. https://doi.org/10.3389/fimmu.2020.575597.

24. Rwandamuriye, F.X., Wang, T., Zhang, H., Elaskalani, O., Kuster, J., Ye, X., Vitali, B., Schreurs, J., Orozco Morales, M.L., Norret, M., et al. (2024). Local therapy with combination TLR agonists stimulates systemic anti-tumor immunity and sensitizes tumors to immune checkpoint blockade. OncoImmunology *13*, 2395067. https://doi.org/10.1080/2162402X.2024.2395067.

25. Santollani, L., Maiorino, L., Zhang, Y.J., Palmeri, J.R., Stinson, J.A., Duhamel, L.R., Qureshi, K., Suggs, J.R., Porth, O.T., Pinney, W., 3rd, et al. (2024). Local delivery of cell surface-targeted immunocytokines programs systemic antitumor immunity. Nat. Immunol. *25*, 1820–1829. https://doi.org/10.1038/s41590-024-01925-7.

26. Hamouda, A.E.I., Filtjens, J., Brabants, E., Kancheva, D., Debraekeleer, A., Brughmans, J., Jacobs, L., Bardet, P.M.R., Knetemann, E., Lefesvre, P., et al. (2024). Intratumoral delivery of lipid nanoparticle-formulated mRNA encoding IL-21, IL-7, and 4-1BBL induces systemic anti-tumor immunity. Nat. Commun. *15*, 10635. https://doi.org/10.1038/s41467-024-54877-9.

27. Lucas, M.L., Heller, L., Coppola, D., and Heller, R. (2002). IL-12 plasmid delivery by in vivo electroporation for the successful treatment of established subcutaneous B16.F10 melanoma. Mol. Ther. *5*, 668–675. https://doi.org/10.1006/mthe.2002.0601.

28. Shi, G., Edelblute, C., Arpag, S., Lundberg, C., and Heller, R. (2018). IL-12 Gene Electrotransfer Triggers a Change in Immune Response within Mouse Tumors. Cancers *10*, 498. https://doi.org/10.3390/cancers10120498.

29. Znidar, K., Bosnjak, M., Cemazar, M., and Heller, L.C. (2016). Cytosolic DNA Sensor Upregulation Accompanies DNA Electrotransfer in B16.F10 Melanoma Cells. Mol. Ther. Nucleic Acids *5*, e322. https://doi.org/10.1038/mtna.2016.34.

30. Melero, I., Quetglas, J.I., Reboredo, M., Dubrot, J., Rodriguez-Madoz, J.R., Mancheño, U., Casales, E., Riezu-Boj, J.I., Ruiz-Guillen, M., Ochoa, M.C., et al. (2015). Strict requirement for vector-induced type I interferon in efficacious antitumor responses to virally encoded IL12. Cancer Res. *75*, 497–507. https://doi.org/10.1158/0008-5472.CAN-13-3356.

31. Algazi, A.P., Twitty, C.G., Tsai, K.K., Le, M., Pierce, R., Browning, E., Hermiz, R., Canton, D.A., Bannavong, D., Oglesby, A., et al. (2020). Phase II Trial of IL-12 Plasmid Transfection and PD-1 Blockade in Immunologically Quiescent Melanoma. Clin. Cancer Res. *26*, 2827–2837. https://doi.org/10.1158/1078-0432.CCR-19-2217.

32. Bhatia, S., Longino, N.V., Miller, N.J., Kulikauskas, R., Iyer, J.G., Ibrani, D., Blom, A., Byrd, D.R., Parvathaneni, U., Twitty, C.G., et al. (2020). Intratumoral Delivery of Plasmid IL12 Via Electroporation Leads to Regression of Injected and Noninjected Tumors in Merkel Cell Carcinoma. Clin. Cancer Res. *26*, 598–607. https://doi.org/10.1158/1078-0432.CCR-19-0972.

33. Donate, A., Burcus, N., Schoenbach, K., and Heller, R. (2015). Application of increased temperature from an exogenous source to enhance gene electrotransfer. Bioelectrochemistry *103*, 120–123. https://doi.org/10.1016/j.bioelechem.2014.08.007.

34. Edelblute, C., Mangiamele, C., and Heller, R. (2021). Moderate Heat-Assisted Gene Electrotransfer as a Potential Delivery Approach for Protein Replacement Therapy through the Skin. Pharmaceutics *13*, 1908. https://doi.org/10.3390/pharmaceutics13111908.

35. Bulysheva, A., Hornef, J., Edelblute, C., Jiang, C., Schoenbach, K., Lundberg, C., Malik, M.A., and Heller, R. (2019). Coalesced thermal and electrotransfer mediated delivery of plasmid DNA to the skin. Bioelectrochemistry *125*, 127–133. https://doi.org/10.1016/j.bioelechem.2018.10.004.

36. Atkins, R.M., Fawcett, T.J., Gilbert, R., Hoff, A.M., Connolly, R., Brown, D.W., Llewellyn, A.J., and Jaroszeski, M.J. (2017). Impedance spectroscopy as an indicator for successful in vivo electric field mediated gene delivery in a murine model. Bioelectrochemistry *115*, 33–40. https://doi.org/10.1016/j.bioelechem.2017.01.004.

37. Atkins, R.M., Fawcett, T.J., Gilbert, R., Hoff, A.M., Connolly, R., Brown, D.W., and Jaroszeski, M.J. (2021). Real-time impedance feedback to enhance cutaneous gene electrotransfer in a murine skin model. Bioelectrochemistry *142*, 107885. https://doi.org/10.1016/j.bioelechem.2021.107885.

38. Mehrara, E., Forssell-Aronsson, E., Ahlman, H., and Bernhardt, P. (2009). Quantitative analysis of tumor growth rate and changes in tumor marker level: specific growth rate versus doubling time. Acta Oncol. *48*, 591–597. https://doi.org/10.1080/02841860802616736.

39. Shirley, S.A., Lundberg, C.G., Li, F., Burcus, N., and Heller, R. (2015). Controlled gene delivery can enhance therapeutic outcome for cancer immune therapy for melanoma. Curr. Gene Ther. *15*, 32–43. https://doi.org/10.2174/1566523214666141121111630.

40. Nguyen, K., Hignett, E., and Khachemoune, A. (2020). Current and emerging treatment options for metastatic melanoma: a focused review. Dermatol. Online J. *26*, 13030/qt24g3k7z5.

41. Schadendorf, D., Livingstone, E., and Zimmer, L. (2019). Treatment in metastatic melanoma-time to re-think. Ann. Oncol. *30*, 501–503. https://doi.org/10.1093/annonc/mdz050.

42. Chapman, P.B., Einhorn, L.H., Meyers, M.L., Saxman, S., Destro, A.N., Panageas, K.S., Begg, C.B., Agarwala, S.S., Schuchter, L.M., Ernstoff, M.S., et al. (1999). Phase III multicenter randomized trial of the Dartmouth regimen versus dacarbazine in patients with metastatic melanoma. J. Clin. Oncol. *17*, 2745–2751. https://doi.org/10.1200/JCO.1999.17.9.2745.

43. Middleton, M.R., Grob, J.J., Aaronson, N., Fierlbeck, G., Tilgen, W., Seiter, S., Gore, M., Aamdal, S., Cebon, J., Coates, A., et al. (2000). Randomized phase III study of temozolomide versus dacarbazine in the treatment of patients with advanced metastatic malignant melanoma. J. Clin. Oncol. *18*, 158–166. https://doi.org/10.1200/JCO.2000.18.1.158.

44. Avril, M.F., Aamdal, S., Grob, J.J., Hauschild, A., Mohr, P., Bonerandi, J.J., Weichenthal, M., Neuber, K., Bieber, T., Gilde, K., et al. (2004). Fotemustine compared with dacarbazine in patients with disseminated malignant melanoma: a phase III study. J. Clin. Oncol. *22*, 1118–1125. https://doi.org/10.1200/JCO.2004.04.165.

45. Knight, A., Karapetyan, L., and Kirkwood, J.M. (2023). Immunotherapy in Melanoma: Recent Advances and Future Directions. Cancers *15*, 1106. https://doi.org/10.3390/cancers15041106.

46. Leonard, J.P., Sherman, M.L., Fisher, G.L., Buchanan, L.J., Larsen, G., Atkins, M.B., Sosman, J.A., Dutcher, J.P., Vogelzang, N.J., and Ryan, J.L. (1997). Effects of single-dose interleukin-12 exposure on interleukin-12-associated toxicity and interferon-gamma production. Blood *90*, 2541–2548.

47. Heller, L., Pottinger, C., Jaroszeski, M.J., Gilbert, R., and Heller, R. (2000). In vivo electroporation of plasmids encoding GM-CSF or interleukin-2 into existing B16 melanomas combined with electrochemotherapy induces longterm antitumour immunity. Melanoma Res. *10*, 577–583.

48. Heller, L.C., Ingram, S.F., Lucas, M.L., Gilbert, R.A., and Heller, R. (2002). Effect of electrically mediated intratumor and intramuscular delivery of a plasmid encoding IFN alpha on visible B16 mouse melanomas. Technol. Cancer Res. Treat. *1*, 205–209. https://doi.org/10.1177/153303460200100305.

49. Marrero, B., Shirley, S., and Heller, R. (2014). Delivery of Interleukin-15 to B16 Melanoma by Electroporation Leads to Tumor Regression and Long-term Survival. Technol. Cancer Res. Treat. *13*, 551–560. https://doi.org/10.7785/tcrtexpress.2013.600252.

50. Heller, L., Merkler, K., Westover, J., Cruz, Y., Coppola, D., Benson, K., Daud, A., and Heller, R. (2006). Evaluation of toxicity following electrically mediated interleukin-12 gene delivery in a B16 mouse melanoma model. Clin. Cancer Res. *12*, 3177–3183. https://doi.org/10.1158/1078-0432.CCR-05-2727.

51. Algazi, A., Bhatia, S., Agarwala, S., Molina, M., Lewis, K., Faries, M., Fong, L., Levine, L.P., Franco, M., Oglesby, A., et al. (2020). Intratumoral delivery of tavokinogene telseplasmid yields systemic immune responses in metastatic melanoma patients. Ann. Oncol. *31*, 532–540. https://doi.org/10.1016/j.annonc.2019.12.008.

52. Heller, L.C., and Coppola, D. (2002). Electrically mediated delivery of vector plasmid DNA elicits an antitumor effect. Gene Ther. *9*, 1321–1325. https://doi.org/10.1038/sj.gt.3301802.

53. Heller, L., Todorovic, V., and Cemazar, M. (2013). Electrotransfer of single-stranded or double-stranded DNA induces complete regression of palpable B16.F10 mouse melanomas. Cancer Gene Ther. *20*, 695–700. https://doi.org/10.1038/cgt.2013.71.

54. Bosnjak, M., Jesenko, T., Kamensek, U., Sersa, G., Lavrencak, J., Heller, L., and Cemazar, M. (2018). Electrotransfer of Different Control Plasmids Elicits Different Antitumor Effectiveness in B16. Cancers (Basel). *10*, 37. https://doi.org/10.3390/cancers10020037.

55. Shi, G., Synowiec, J., Singh, J., and Heller, R. (2024). Modification of the tumor microenvironment enhances immunity with plasmid gene therapy. Cancer Gene Ther. *31*, 641–648. https://doi.org/10.1038/s41417-024-00728-0.

OMTON, Volume *33*

Supplemental information

Effective gene immunotherapy for melanoma utilizing an advanced *in vivo* electrotransfer system

Loree C. Heller, Julie S. Singh, Jody C. Synowiec, Pavan Kumar Cherukuri, Nhat Phan, Guilan Shi, Mark J. Jaroszeski, Alex Otten, and Richard Heller

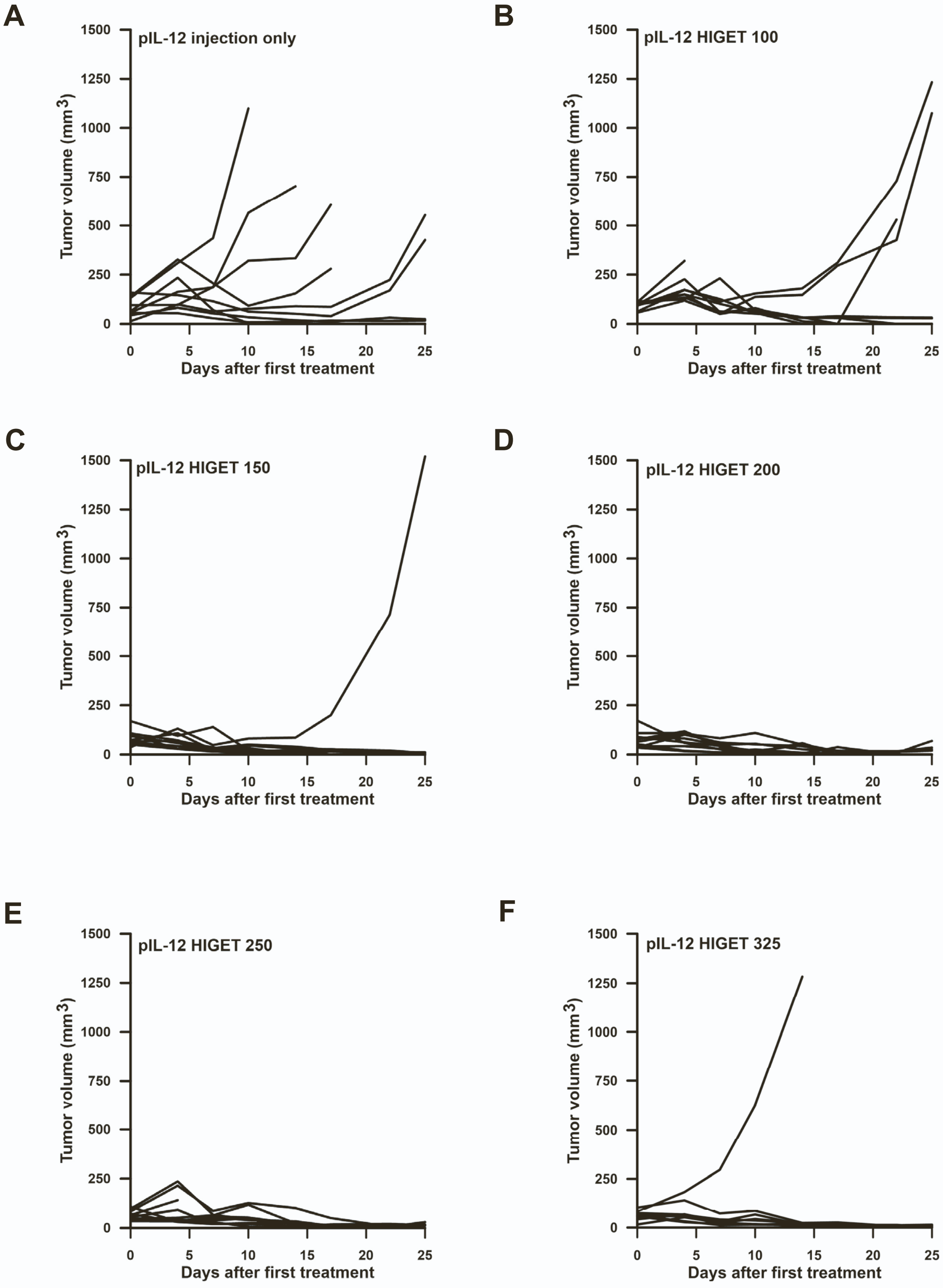

Figure S1. Individual tumor growth following **A,** pIL-12 injection only; **B,** pIL-12 injection plus HIGET 100 pulses; **C,** pIL-12 injection plus HIGET 150 pulses; **D,** pIL-12 injection plus HIGET 200 pulses; **E,** pIL-12 injection plus HIGET 250 pulses; **F** pIL-12 injection plus HIGET 325 pulses.

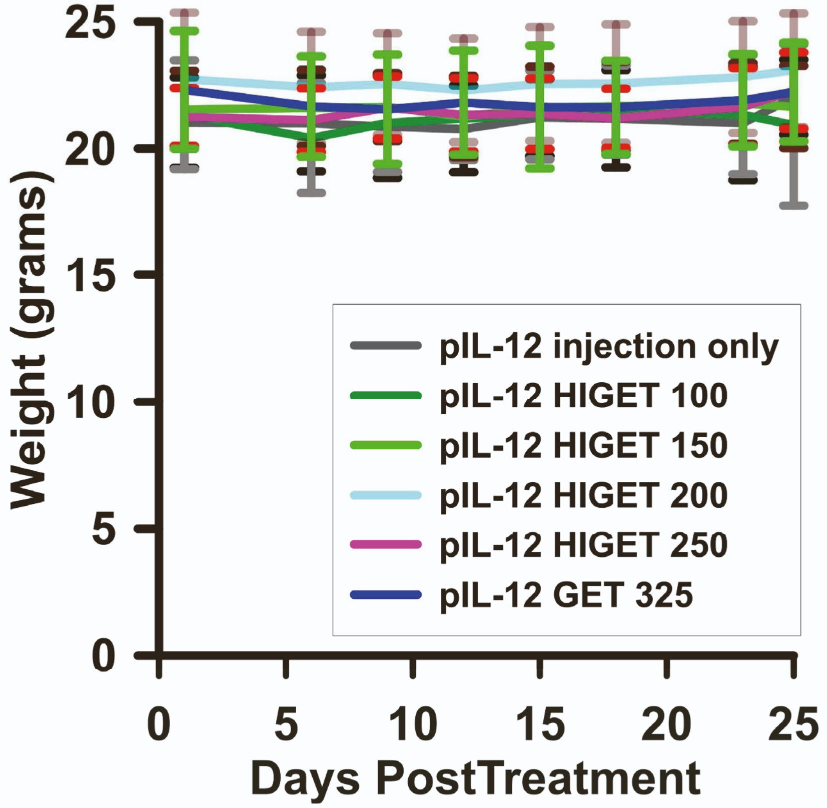

Figure S2. Body weight was not affected by HIGET delivery protocol. Numbers (100, 150, 200, 250, 325) indicate applied voltage (Table 1). Repeated measures ANOVA analysis indicated that weight did not significantly change across all dates and groups. pIL-12, pUMVC3-mIL-12; HIGET, heat and impedance gene electrotransfer.

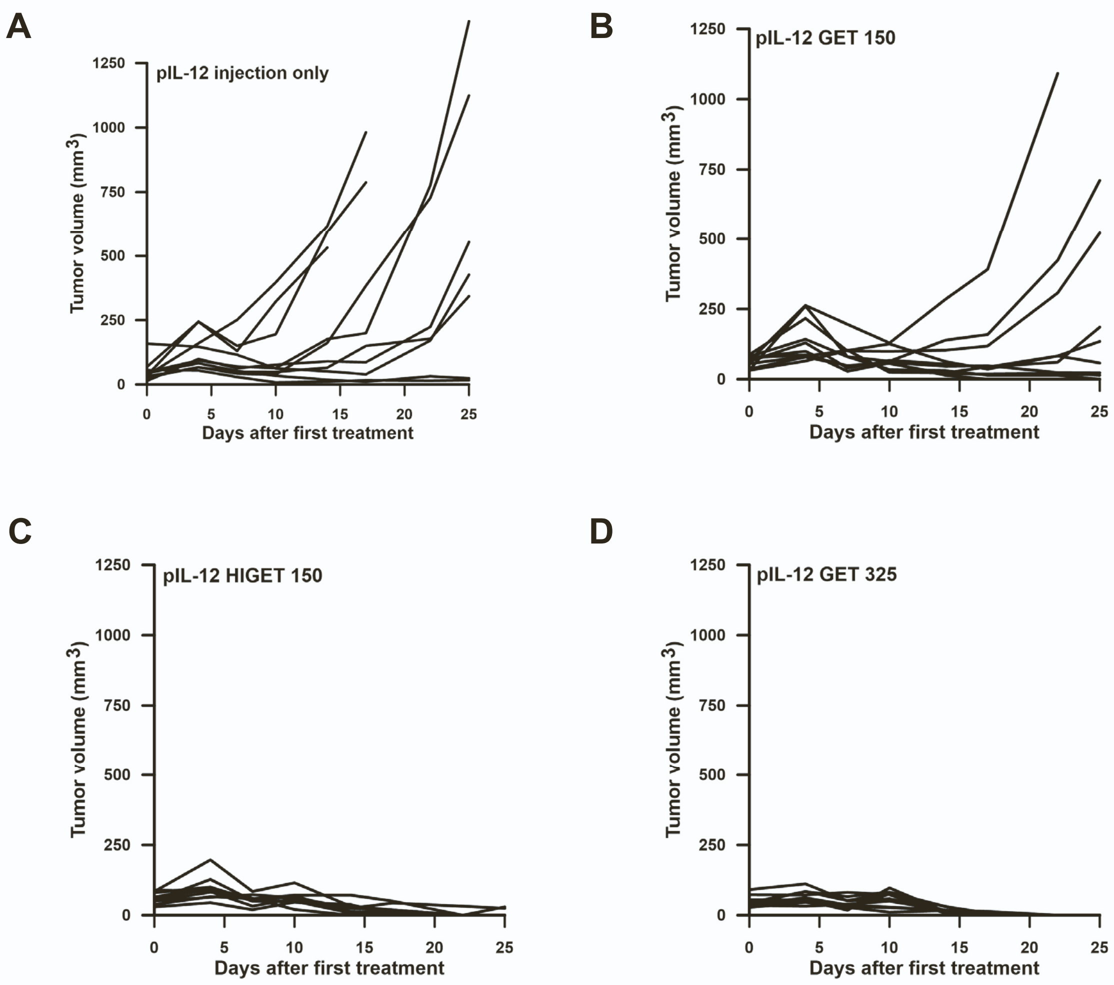

Figure S3. Individual tumor growth following **A,** pIL-12 injection only; **B,** pIL-12 injection plus GET 150 pulses; **C,** pIL-12 injection plus HIGET 150 pulses; **D,** pIL-12 injection plus GET 325 pulses.

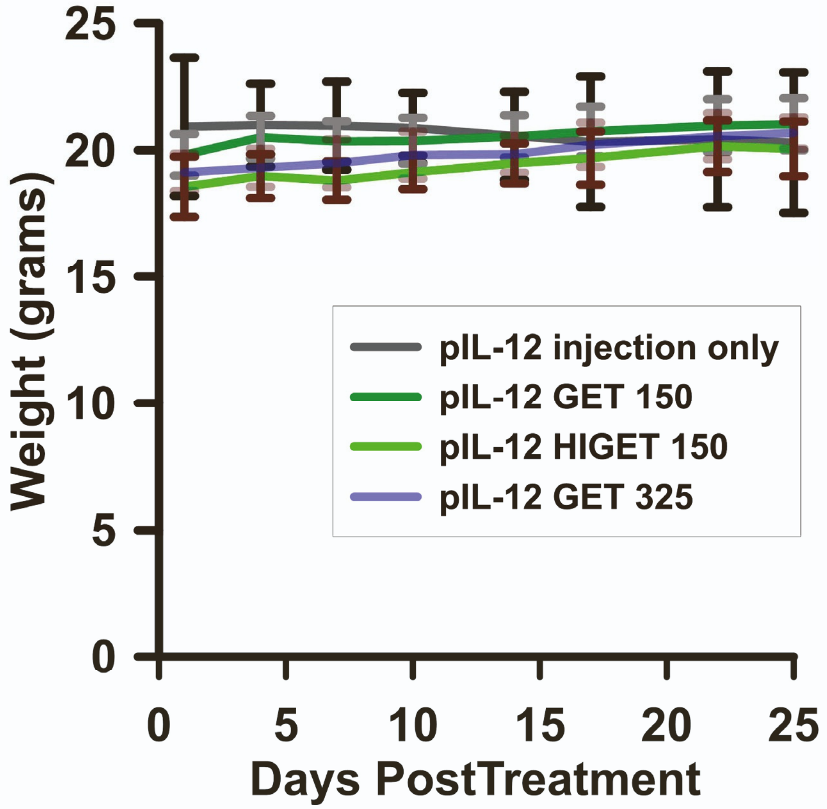

Figure S4. GET or HIGET plasmid delivery did not affect body weight. Numbers (150, 325) indicate applied voltage. Repeated measures ANOVA analysis indicated that weight did not significantly change across all dates and groups. pIL-12, pUMVC3-mIL12; GET, gene electrotransfer; HIGET, heat-and-impedance gene electrotransfer.
